# Definitive radiotherapy dose escalation with chemotherapy for treating non-metastatic oesophageal cancer

**DOI:** 10.1038/s41598-018-31302-y

**Published:** 2018-08-27

**Authors:** Chao-Yueh Fan, Yu-Fu Su, Wen-Yen Huang, Hsing-Lung Chao, Kuen-Tze Lin, Chun-Shu Lin

**Affiliations:** Department of Radiation Oncology, Tri-Service General Hospital, National Defense Medical Center, Taipei, Taiwan

## Abstract

The locoregional failure rate remains high after concurrent chemoradiotherapy with standard-dose radiotherapy (RT, 50–50.4 Gy) for oesophageal cancer (EC). This retrospective study evaluated whether RT dose escalation was effective among 115 consecutive patients with non-metastatic EC (July 2003 to November 2016). Forty-four patients received an RT dose of <66 Gy and 71 patients received ≥66 Gy, with most patients receiving concurrent cisplatin plus fluorouracil. The median follow-up was 12 months for all patients (52 months for 18 surviving patients). The ≥66 Gy group had significantly higher 3-year rates of overall survival (17.9% vs. 32.1%, p = 0.026) and local progression-free survival (46.1% vs. 72.1%, p = 0.005), but not disease progression-free survival (11.4% vs. 21.9%, p = 0.059) and distant metastasis-free survival (49% vs. 52.6%, p = 0.852). The ≥66 Gy group also had significantly better 5-year overall survival compared with 41.4–65.9 Gy. The only significant difference in treatment-related toxicities involved acute dermatitis (7% vs. 28%, p = 0.009). Inferior overall survival was associated with poor performance status, clinical N2–3 stage and not receiving maintenance chemotherapy. In conclusion, patients with inoperable EC experienced better survival outcomes and acceptable toxicities if they received higher dose RT (≥66 Gy) rather than lower dose RT (<66 Gy).

## Introduction

Major advances in surgery, radiotherapy (RT), and chemotherapy have established multimodal curative treatment options for oesophageal cancer (EC)^1^. Definitive concurrent chemoradiotherapy (CCRT) is the preferred treatment for inoperable patients, cases with unresectable EC, or patients who decline surgery^2^. However, the locoregional failure rate remains high (41–50%) after definitive CCRT using a radiation dose of 50.4 Gy for EC, with the vast majority of failures involving the primary tumour (86–90%)^3,4^. Thus, the standard radiation dose (50–50.4 Gy)^1^ may be suboptimal, and some studies have suggested that a higher RT dose to the primary tumour might improve local control and survival^5,6^.

The standard radiation dose for definitive CCRT is based on the INT-0123 trial, which compared high and standard doses (64.8 Gy vs. 50.4 Gy) for locally advanced EC^7^, and was prematurely closed because of multiple deaths in the high-dose group and no differences in survival or local control. However, 7 of the 11 treatment-related deaths in the high-dose group occurred before the radiation dose reached 50.4 Gy, and the high-dose group also received a significantly lower dose of fluorouracil, which might have affected the outcomes in that group. Moreover, the investigators used a conventional two-dimensional technique to deliver RT, rather than relatively safer modern RT techniques, such as three-dimensional conformal radiotherapy (3DCRT), intensity-modulated radiotherapy (IMRT), or volumetric modulated arc therapy (VMAT). Several dosimetric studies have explored the use of IMRT or VMAT to maintain the therapeutic ratio by administering a high RT dose to the tumour while minimizing the dose to the surrounding organs^8–11^. This single-centre retrospective study aimed to evaluate the clinical benefit of high-dose RT using modern techniques for treating EC.

## Results

Table 1 shows the baseline characteristics of the groups that received an RT dose of <66 Gy (n = 44; “the <66 Gy group”) or ≥66 Gy (n = 71; “the ≥66 Gy group”). The median patient age at diagnosis was 61 years (range: 32–86 years). The patients predominantly were male (93%), and had squamous cell carcinoma (95%). The median tumour length was 7 cm (range: 2–20 cm). The main reason for not undergoing surgery was an unresectable tumour (59%). Eighty-five patients (74%) received maintenance chemotherapy. The RT techniques included VMAT (49 patients, 43%), IMRT (43 patients, 37%), and 3DCRT (23 patients, 20%).

**Table 1:** Comparing the patients’ clinical characteristics.

Characteristics	Number of patients (%)			
	All (n = 115)	RT dose < 66 Gy (n = 44)	RT dose ≥ 66 Gy (n = 71)	p value
Age, years				
<60	54 (47)	19 (43)	35 (49)	0.523
≥60	61 (53)	25 (57)	36 (51)	
Median, range	61, 32-86	62, 32-85	60, 39-86	
Sex				
Male	107 (93)	40 (91)	67 (94)	0.479
Female	8 (7)	4 (9)	4 (6)	
Performance				
ECOG 0	52 (45)	13 (29)	39 (55)	**0**.**027**
ECOG 1	52 (45)	25 (57)	27 (38)	
ECOG 2	11 (10)	6 (14)	5 (7)	
Tumor histology				
SqCC	110 (95)	42 (96)	68 (96)	0.929
Adenocarcinoma	3 (3)	1 (2)	2 (3)	
Other carcinoma	2 (2)	1 (2)	1 (1)	
Tumor grade				
GX, G1	14 (12)	4 (9)	10 (14)	0.701
G2	37 (32)	14 (32)	23 (32)	
G3	64 (56)	26 (59)	38 (54)	
Tumor location				
Cervical	16 (14)	5 (11)	11 (15)	0.902
Upper third	25 (22)	10 (23)	15 (21)	
Middle third	38 (33)	14 (32)	24 (34)	
Lower third/EGJ	36 (31)	15 (34)	21 (30)	
Tumor length				
≤5 cm	45 (39)	17 (39)	28 (39)	0.932
>5 cm	70 (61)	27 (61)	43 (61)	
Median, range	7, 2-20	7, 2-14	6, 2-20	
Clinical T stage				
T1-2	25 (22)	8 (18)	17 (24)	0.761
T3	60 (52)	24 (55)	36 (51)	
T4a	10 (9)	3 (7)	7 (10)	
T4b	20 (17)	9 (20)	11 (15)	
Clinical N stage				
N0	29 (25)	12 (27)	17 (24)	0.222
N1	38 (33)	18 (41)	29 (28)	
N2	27 (24)	6 (14)	21 (30)	
N3	21 (18)	8 (18)	13 (18)	
Reason for no surgery				
Unresectable tumor	68 (59)	23 (52)	45 (63)	0.476
Patient’s choice	19 (17)	9 (21)	10 (14)	
Medically inoperable	28 (24)	12 (27)	16 (23)	
Chemotherapy regimen				0.495
Cisplatin + 5-FU	95 (83)	35 (80)	60 (85)	
Cisplatin or 5-FU monotherapy	20 (17)	9 (20)	11 (16)	
Maintenance chemotherapy				0.124
Yes	85 (74)	29 (66)	56 (79)	
No	30 (26)	15 (34)	15 (21)	
RT technique				
VMAT	49 (43)	15 (34)	34 (48)	0.292
IMRT	43 (37)	20 (46)	23 (32)	
3DCRT	23 (20)	9 (20)	14 (20)	

Abbreviations: RT, radiotherapy; ECOG, Eastern Cooperative Oncology Group; SqCC, squamous cell carcinoma; G, grade; EGJ, esophagogastric junction; CCRT, concurrent chemoradiotherapy; 5-FU, 5-fluorouracil; VMAT, volumetric modulated arc therapy; IMRT, intensity-modulated radiotherapy; 3DCRT, three-dimensional conformal radiotherapy.

The median ages were 62 years (range: 32–85 years) for the <66 Gy group and 60 years (range: 39–86 years) for the ≥66 Gy group (p = 0.523). The ≥66 Gy group had a significantly higher proportion of patients with a good performance status (Eastern Cooperative Oncology Group performance status [ECOG PS] of 0) (p = 0.027). There were no significant differences between the <66 Gy and ≥66 Gy groups in terms of their sex, tumour histology, grade, location, length, clinical T stage, clinical N stage, reasons for no surgery, chemotherapy regimens, maintenance chemotherapy status, and RT technique.

The median follow-up durations were 12 months (range: 2–103 months) for all patients and 52 months (range: 12–100 months) for the 18 surviving patients. The 3-year and 5-year overall survival (OS) rates for all patients were 26.6% and 18.4%, respectively. The 3-year rates of disease progression-free survival (DPFS), local progression-free survival (LPFS), and distant metastasis-free survival (DMPFS) were 17.8%, 62.8%, and 51.5%, respectively.

**Figure 1:**
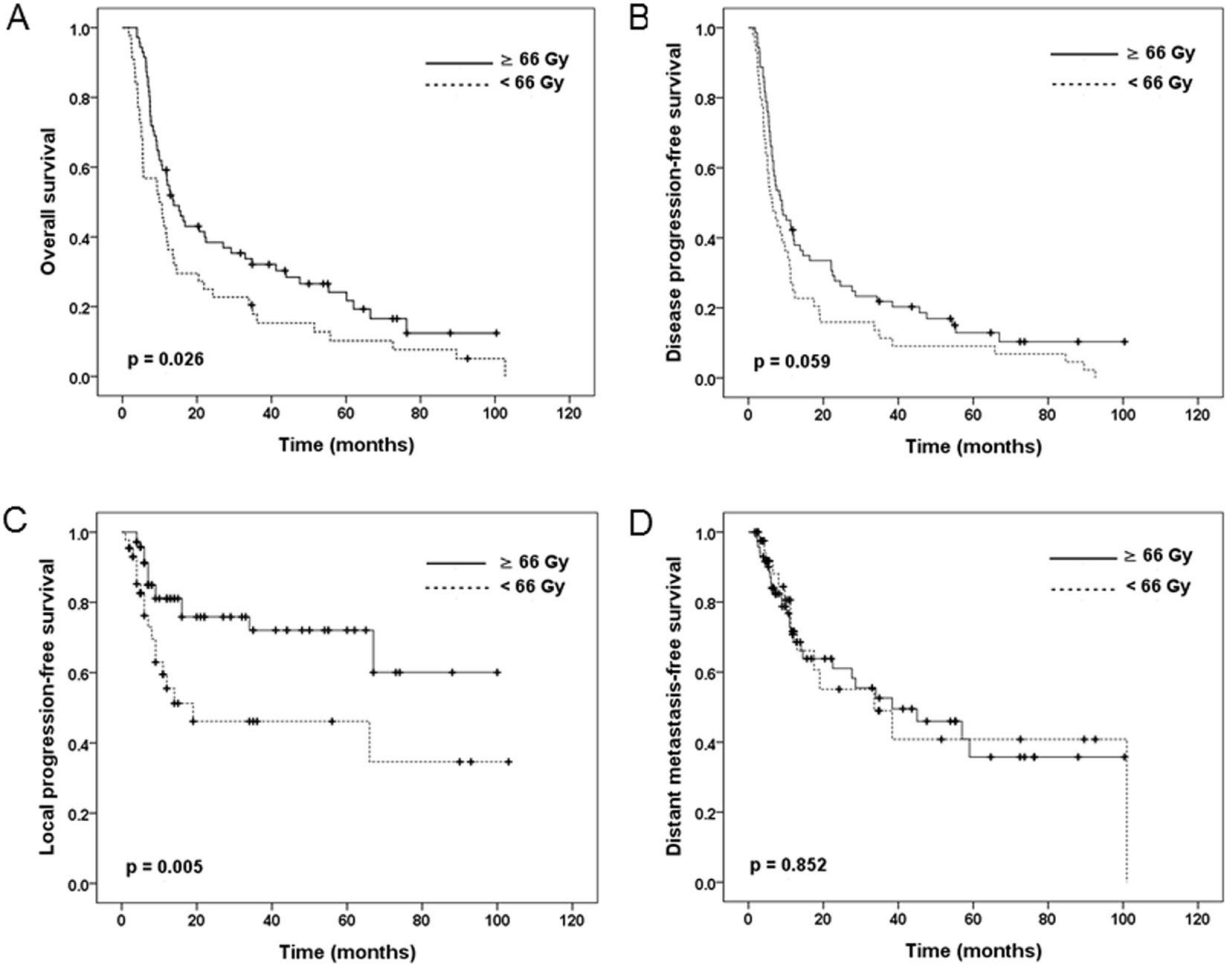
Kaplan-Meier estimates of (**A**) overall survival, (**B**) disease progression-free survival, (**C**) local progression-free survival, and (**D**) distant metastasis-free survival according to radiation dose.

Table 2 shows the results of the univariate analyses for OS, DPFS, LPFS, and DMPFS. The comparisons of the <66 Gy and ≥66 Gy groups revealed significant differences in the 3-year rates of OS (117.9% vs. 32.1%, p = 0.026) and LPFS (46.1% vs. 72.1%, p = 0.005), but not in DPFS (11.4% vs. 21.9%, p = 0.059) and DMPFS (49% vs. 52.6%, p = 0.852). The median OS values in the ≥66 Gy and <66 Gy groups were 13 months and 10.2 months, respectively, which corresponded to 5-year OS rates of 24.1% and 10.2%, respectively. The survival curves of each group are plotted in Fig. 1. In the univariate analyses, poor OS was associated with older age (≥60 years), ECOG PS of 1–2, tumour length of >5 cm, clinical N2–3 stage, and not receiving maintenance chemotherapy.

**Table 2:** Univariate predictors of overall survival, disease progression-free survival, local progression-free survival, and distant metastasis-free survival.

Variable	No.	OS		DPFS		LPFS		DMPFS	
		3 year (%)	p value	3 year (%)	p value	3 year (%)	p value	3 year (%)	p value
All patients	115	26.6		17.8		62.8		51.5	
Age, years			**0.027**		0.355		0.914		0.306
<60	54	32.1		20.4		60.5		60.6	
≥60	61	22.0		15.5		65.1		42.8	
Sex			0.721		0.404		0.731		0.225
Male	107	26.6		17.3		63.2		49.9	
Female	8	25.0		25.0		60.0		83.3	
Performance			**0.003**		**0.012**		**0.01**		0.130
ECOG 0	52	35.7		26.9		73.5		65.1	
ECOG 1-2	63	18.9		10.1		53.2		35.8	
Tumor histology			0.152		0.445		0.406		0.420
SqCC	110	27.8		18.6		62.0		51.6	
Adenocarcinoma	3	0		0		0		—	
Tumor grade			0.947		0.613		0.206		0.181
GX/G1-2	51	30.5		18.6		67.4		61.7	
G3	64	23.5		17.2		59.0		44.7	
Tumor location			0.574		0.222		0.695		0.701
Cervical/upper third	41	32.4		26.0		64.7		54.6	
Middle/lower third/EGJ	74	23.4		13.5		61.0		49.2	
Tumor length			**0.038**		**0.006**		0.132		**0.011**
≤5 cm	45	35.3		28.0		70.7		67.8	
>5 cm	70	21.0		11.4		56.3		39.8	
Clinical T stage			0.129		0.648		0.308		0.161
T1-2	25	37.4		20.0		62.5		66.0	
T3-4	90	23.5		17.2		62.2		47.1	
Clinical N stage			**0.01**		**0.004**		0.463		<**0.001**
N0-1	67	30.7		22.4		64.4		64.5	
N2-3	48	21.1		11.4		61.6		31.3	
Reason for no surgery			0.734		0.811		0.424		0.768
Unresectable tumor	68	24.3		16.9		65.6		51.4	
Patient’s choice	19	47.4		31.6		61.0		72.7	
Medically inoperable	28	17.9		10.7		61.7		35.2	
RT technique			0.132		**0.039**		0.290		0.153
VMAT/IMRT	92	24.7		13.5		57.6		46.2	
3DCRT	23	34.8		34.8		79.3		69.5	
Maintenance chemotherapy			**0.001**		**0.009**		**0.034**		0.115
Yes	85	31.4		23.1		68.2		58.6	
No	30	12.5		3.3		43.8		21.3	
RT dose			**0.026**		0.059		**0.005**		0.852
<66 Gy	44	17.9		11.4		46.1		49.0	
≥66 Gy	71	32.1		21.9		72.1		52.6	

Abbreviations: OS, overall survival; DPFS, disease progression-free survival; LPFS, local progression-free survival; DMPFS, distant metastasis-free survival; ECOG, Eastern Cooperative Oncology Group; SqCC, squamous cell carcinoma; G, grade; EGJ, esophagogastric junction; CCRT, concurrent chemoradiotherapy; RT, radiotherapy; VMAT, volumetric modulated arc therapy; IMRT, intensity-modulated radiotherapy; 3DCRT, three-dimensional conformal radiotherapy.

Table 3 shows the results of the multivariate analysis, which included the significant factors from the univariate analyses. These results indicated that a RT dose of ≥66 Gy independently predicted better OS (hazard ratio [HR]: 0.646, 95% confidence interval [CI]: 0.425–0.982), and better LPFS (HR: 0.432, 95% CI: 0.216–0.865), but not better DPFS (HR: 0.727, 95% CI: 0.485–1.091) or DMPFS (HR: 1.044, 95% CI: 0.524–2.079). Poor OS was also independently associated with ECOG PS of 1–2, clinical N2–3 stage, and not receiving maintenance chemotherapy. Patients with ECOG PS of 1–2 independently had inferior DPFS, and LPFS. Advanced clinical N stage independently predicted inferior DPFS and DMPFS.

**Table 3:** Prognostic factors in multivariate analysis of overall survival, disease progression-free survival, local progression-free survival and distant metastasis-free survival.

	OS		DPFS		LPFS		DMPFS	
Variable	HR (95% CI)	p value	HR (95% CI)	p value	HR (95% CI)	p value	HR (95% CI)	p value
Age, years								
≥60	1.295 (0.830, 2.021)	0.255	0.999 (0.655, 1.525)	0.998	0.679 (0.325, 1.421)	0.305	1.191 (0.611, 2.321)	0.608
Performance								
ECOG 1-2	1.602 (1.022, 2.510)	**0**.**040**	1.545 (1.009, 2.366)	**0**.**046**	2.495 (1.139, 5.467)	**0**.**022**	1.727 (0.894, 3.336)	0.104
Tumor length								
>5 cm	1.372 (0.870, 2.164)	0.173	1.489 (0.959 2.312)	0.076	1.502 (0.696, 3.242)	0.300	1.926 (0.930, 3.988)	0.077
Clinical N stage								
N2-3	1.645 (1.060, 2.551)	**0**.**026**	1.673 (1.099, 2.546)	**0**.**016**	1.384 (0.659, 2.905)	0.391	2.741 (1.423, 5.278)	**0**.**003**
Maintenance chemotherapy								
No	1.780 (1.114, 2.845)	**0.016**	1.504 (0.954, 2.369)	0.079	1.882 (0.886, 3.997)	0.100	1.406 (0.672, 2.943)	0.366
**RT dose**								
≥66 Gy	0.646 (0.425, 0.982)	**0**.**041**	0.727 (0.485, 1.091)	0.123	0.432 (0.216, 0.865)	**0**.**018**	1.044 (0.524, 2.079)	0.903

Abbreviations: OS, overall survival; DPFS, disease progression-free survival; LPFS, local progression-free survival; DMPFS, distant metastasis-free survival; HR, hazard ratio; CI, confidence interval; ECOG, Eastern Cooperative Oncology Group; RT, radiotherapy.

**Figure 2:**
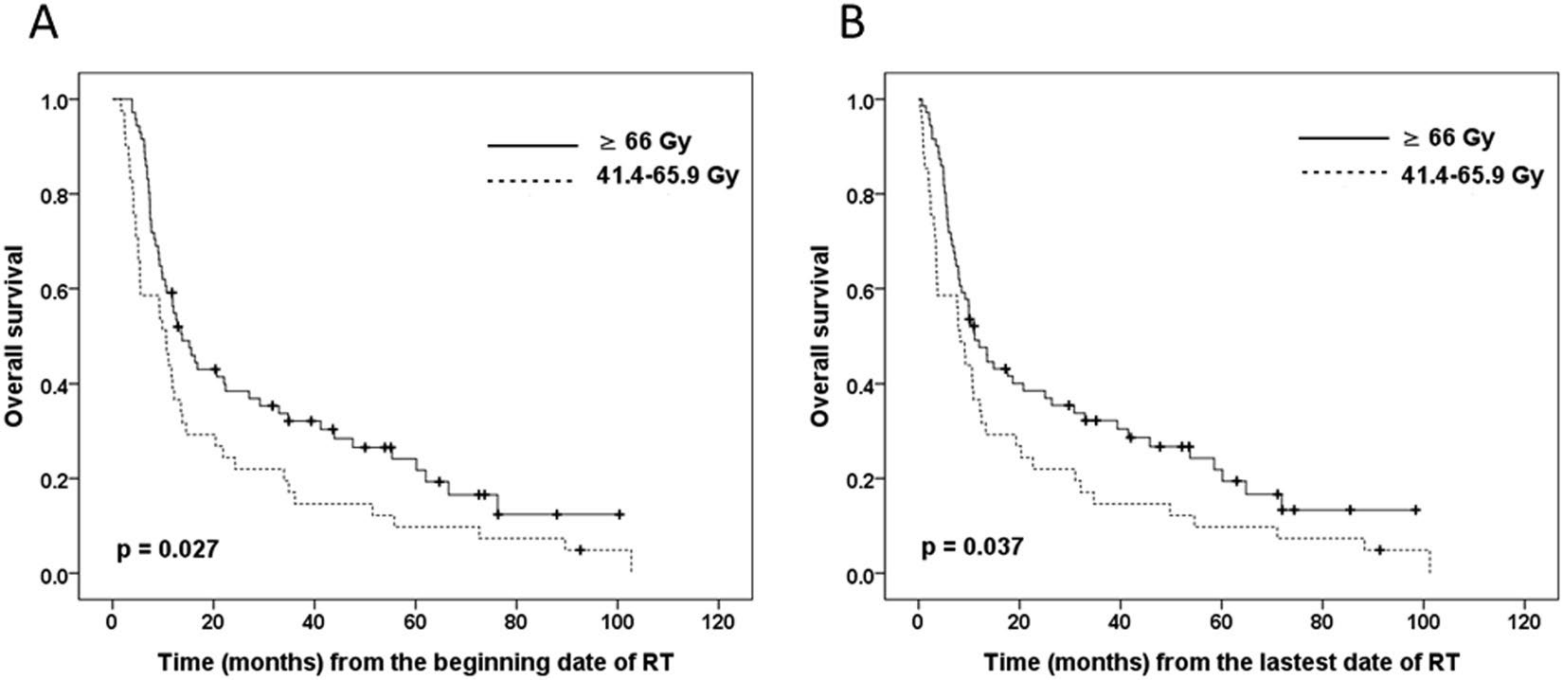
Kaplan-Meier curves of overall survival estimated from the (**A**) beginning date or (**B**) the latest date of RT stratified based on radiation doses of 41.4–65.9 Gy versus ≥66 Gy.

**Figure 3:**
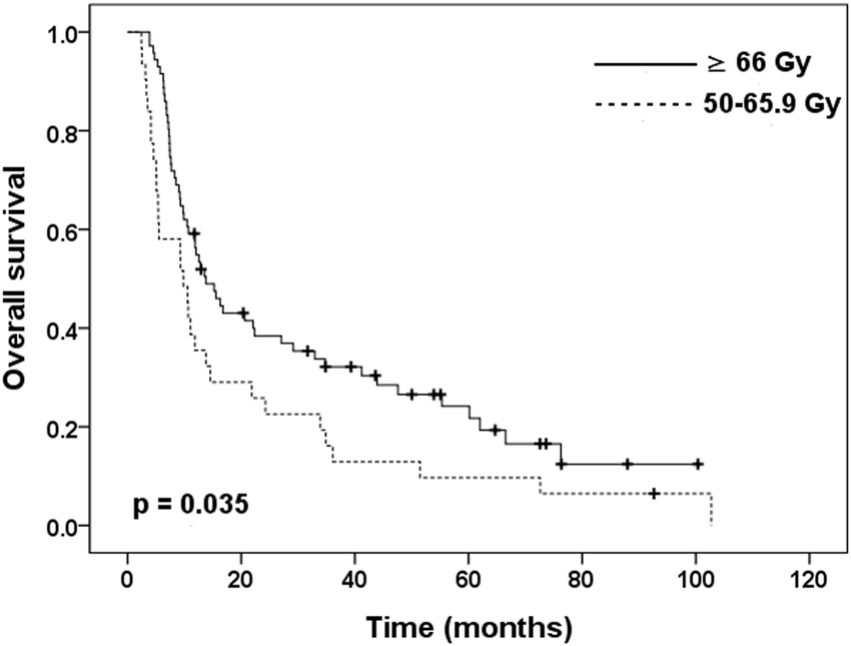
Kaplan-Meier curves of overall survival stratified based on radiation doses of 50–65.9 Gy versus ≥66 Gy.

The subgroup analysis excluded 3 patients who received an RT dose of <41.4 Gy, but still revealed a significantly higher 3-year OS rate in the ≥66 Gy group than in the 41.4–65.9 Gy group (32.1% vs. 17.1%, p = 0.027) (Fig. 2A). We also calculated the OS from the latest date of RT to death or the last follow-up, which still revealed a significantly better 3-year OS rate in the ≥66 Gy group than in the 41.4–65.9 Gy group (32.2% vs. 14.6%, p = 0.037) (Fig. 2B). We further excluded 13 patients with RT dose <50 Gy. The 3-year OS rate was significantly higher in the ≥66 Gy group relative to the 50–65.9 Gy group, with rates of 32.1% and 16.1%, respectively (p = 0.035) (Fig. 3).

The treatment-related toxicities are shown in Table 4. The only significant difference between the <66 Gy and ≥66 Gy groups was observed for acute dermatitis (3 patients [7%] vs. 20 patients [28%], p = 0.009), with a trend towards more acute grade ≥2 dysphagia in the ≥66 Gy group (41% vs. 58%, p = 0.073). No significant differences were observed for acute anaemia (p = 0.120), acute neutropenia (p = 0.605), pneumonitis (p = 0.190), tracheoaortic and tracheoesophageal fistula (p = 0.558), or benign oesophageal stricture (p = 0.431).

**Table 4:** Toxicities according to radiotherapy doses of <66 Gy or ≥ 66 Gy.

	RT dose <66 Gy (n = 44)	RT dose ≥66 Gy (n = 71)	p value
Acute dysphagia			0.073
G1	11 (25%)	12 (17%)	
G2	17 (39%)	34 (48%)	
G3	1 (2%)	7 (10%)	
G4	0	0	
Acute dermatitis			**0.009**
G1	3 (7%)	11 (15%)	
G2	0	8 (11%)	
G3	0	1 (1%)	
G4	0	0	
Acute anemia			0.120
G1	13 (30%)	37 (52%)	
G2	24 (55%)	24 (34%)	
G3	6 (14%)	10 (14%)	
G4	0	0	
Acute neutropenia			0.605
G1	8 (18%)	18 (25%)	
G2	16 (36%)	35 (49%)	
G3	9 (21%)	12 (17%)	
G4	4 (9%)	5 (7%)	
Pneumonitis			0.190
G1	0	9 (13%)	
G2	1 (2%)	5 (7%)	
G3	1 (2%)	0	
G4	2 (5%)	0	
TA or TE fistula			0.558
Yes	8 (18%)	10 (14%)	
Esophageal stricture			0.431
Yes	2 (5%)	6 (9%)	

Abbreviations: RT, radiotherapy; G, grade; TA, tracheoaortic; TE, tracheoesophageal.

## Discussion

The present study investigated the relationship between RT dose and survival among patients with non-metastatic EC who underwent definitive RT with chemotherapy. The results indicate that a total RT dose of ≥66 Gy at the primary tumour was associated with significantly better OS and LPFS than a dose of <66 Gy, and this difference persisted after excluding patients who received an inadequate RT dose (<41.4 Gy or <50 Gy). It is possible that the results were related to the immortal time bias^12^, as we initially evaluated survival from the start date of RT for both groups. However, we also evaluated the survival outcomes from the last date of RT, which eliminated the immortal time bias and revealed that the significant difference in OS persisted between the two groups (Fig. 2B).

Although various studies have evaluated RT dose escalation for treating EC, the potential clinical benefits remain controversial^5,6,13–17^. Brower *et al*. used the National Cancer Data Base in the US to evaluate RT dose escalation for patients with stage I–III EC who underwent CCRT^13^, and reported that higher RT doses (51–54, 55–60, or >60 Gy) did not improve OS relative to a dose of 50–50.4 Gy. However, their cohort only included a limited number of patients who underwent IMRT or 3DCRT (39%), and the predominance of cases with an unknown RT modality (61%) might have influenced the apparent effect of RT dose on survival in the modern era of RT. Recently, Xu *et al*. reported an abstract of a randomized study to evaluate the clinical benefit of high-dose RT using modern techniques^14^. They found no significant differences in local/regional progression-free survival, DPFS, OS and toxicity between the high-dose (60 Gy) and low-dose (50 Gy) group. Chen *et al*. performed a population-based propensity score-matched study using Taiwanese registry data from patients with EC who underwent IMRT or 3DCRT^15^, and reported that an RT dose of ≥60 Gy may provide better 5-year OS than 50–50.4 Gy (22% vs. 14%, p < 0.05). He *et al*. retrospectively reviewed 193 patients with EC who underwent CCRT^16^, and reported that the high-dose group (>50.4 Gy) had a significantly lower local failure rate (17.9% vs. 34.3%, p = 0.024) than the low-dose group (≤50.4 Gy), but there was no significant difference in the 5-year OS rates (41.7% vs. 33.0%, p = 0.617). Cao *et al*. evaluated outcomes among 115 patients with squamous cell carcinoma of the cervical oesophagus who underwent definitive RT with or without chemotherapy^17^, and reported significantly higher rates of distant failure-free survival and OS for their ≥66 Gy group than for their <66 Gy group. Kim *et al*. investigated 236 patients with stage II–III EC who underwent definitive CCRT (<60 Gy for 120 patients and ≥60 Gy for 116 patients)^6^, and reported that the high-dose group experienced significantly better 2-year locoregional control (69.1% vs. 50.3%, p = 0.002), median PFS (16.7 months vs. 11.7 months, p = 0.029), and median OS (35.1 months vs. 22.3 months, p = 0.043). A recent systematic review by Song *et al*. has also indicated that CCRT with an RT dose of >60 Gy provided better tumour response, locoregional control, and OS^5^. Our results also support the association between improved OS and higher RT doses (≥66 Gy), relative to doses of <66 Gy or 41.4–65.9 Gy.

The present study revealed that many radiation oncologists in our department did not follow the NCCN guidelines’ recommendations regarding the RT dose for treating EC, as most patients (96/115 patients) received doses of >50.4 Gy. It provided an opportunity to examine the survival and toxicity effects of escalated RT doses (≥66 Gy) in this setting. Kim *et al*. have reported a positive correlation between RT dose and locoregional control among patients with EC^6^. Furthermore, Lertbutsayanukul *et al*. evaluated 44 patients with locally advanced EC who underwent CCRT (>50 Gy) before esophagectomy^18^, and reported that an RT dose of >60 Gy was associated with better pathological complete remission than lower doses (59.1% vs. 36.4%). Moreover, Welsh *et al*. reported that unresectable EC with gross tumour volume (GTV) failure after CCRT was associated with shorter survival than cases without GTV failure, which suggested that local control helped improve survival^3^. Therefore, we assume that higher RT doses in the present study provided improved local control that translated into improvements in OS.

Regarding treatment-related toxicity, one systematic review has indicated that there were no differences in grade ≥3 acute or late esophagitis between high-dose RT (≥60 Gy) and standard-dose RT^5^. Furthermore, other toxicities are rare and moderately tolerable. Two retrospective studies have also evaluated treatment-related toxicities, with Kim *et al*. reporting no significant differences between the <60 Gy and ≥60 Gy groups^6^. However, relative to a low-dose group (≤50.4 Gy), He *et al*. reported that higher doses (>50.4 Gy) were associated with higher rates of grade 3 skin reactions (12.5% vs. 2.2%, p < 0.001) and oesophageal stricture (32.1% vs. 18.2%, p = 0.037)^16^. In the present study, the only dose-related difference in treatment-related toxicity was observed for acute dermatitis (Table 4), although only 1 patient in the ≥66 Gy group experienced grade 3 acute dermatitis.

The present study has several limitations. The first is the retrospective design and the variable chemotherapy regimens. The second is the different patient characteristics for each group, with the high-dose group having more favourable performance status, which might have influenced the results, although a multivariate Cox proportional hazard model was used to adjust for potential confounding factors. Some may argue that patients in the low-dose group were treated with more palliative intent. However, we analysed the chemotherapy regimens and maintenance chemotherapy status between the <66 Gy and ≥66 Gy groups. No significant differences were found between the 2 groups in terms of chemotherapy regimens and maintenance chemotherapy status, which means that the patients in the low-dose group were not treated with more palliative intent. The third is the regional differences in the histological type of EC, with our cases predominantly involving squamous cell carcinoma (95%). Similar results were reported in the 2015 report by the Taiwan Department of Health^19^, although squamous cell carcinoma is declining in North America and Europe, with concurrent increases in adenocarcinoma of the distal oesophagus and gastroesophageal junction (approximately 70% of oesophageal carcinomas in the US)^20^. Thus, the results of the present study might not be generalizable to regions where the dominant tumour histology is adenocarcinoma. The fourth is the hidden bias which is a common issue for observational study^21^. For example, body weight loss, cachexia, or nutrition status was not controlled in the current study^22^.

In conclusion, the present study’s results suggest that higher RT doses (≥66 Gy), when administered using modern RT technique (3DCRT, IMRT, or VMAT), are feasible for patients with inoperable and non-metastatic EC. In addition, the higher RT doses were associated with significantly better LPFS and OS than for RT doses of <66 Gy. The two groups had similar toxicity incidences and severities, with the only significant differences observed for acute dermatitis, which is a manageable event. Furthermore, inferior OS was independently predicted by poor performance status, clinical N2–3 stage, and not receiving maintenance chemotherapy. Nevertheless, these findings require validation in prospective randomized trials using modern RT techniques.

## Methods

### Data source

We retrospectively evaluated 115 patients with histologically-confirmed EC who underwent definitive RT with chemotherapy at our department between July 2003 and November 2016. All patients had initially undergone a physical examination, chest radiography, barium swallow, chest computed tomography (CT), abdominal sonography, and upper gastrointestinal panendoscopy and/or fluorodeoxyglucose positron emission tomography (PET-CT). Patients were excluded if they had undergone primary surgery or had distant metastasis. Data were collected regarding demographic characteristics, performance status, pathology, imaging results, disease stage, reason for not undergoing surgery, RT, chemotherapy, and follow-up results. Disease staging was based on the 7^th^ version of the American Joint Committee on Cancer guidelines (7^th^ AJCC, 2009). The patients who were diagnosed within 2003–2009 were retrospectively staged in accordance with the 7^th^ AJCC guidelines by reviewing their medical records and images. The retrospective study protocol was approved by the institutional review board of the Tri-Service General Hospital in Taiwan (1-101-05-041). All methods were performed in accordance with the relevant guidelines and regulations.

### Radiotherapy technique

An Elekta linear accelerator was used to deliver RT with 15-MV photons. The GTV contained the primary tumour and metastatic lymph nodes, and was determined using CT, barium swallow, and endoscopy with or without PET-CT. The clinical target volume (CTV) was obtained by expanding the GTV by 3–5 cm at the cranial and caudal margins and by 0.3–2 cm at the transversal margins. The planning target volume included the CTV in the initial phase or the GTV in the boost phase, with additional margins of 0.3–1 cm in all directions to account for organ movement. All patients had undergone VMAT, IMRT, or 3DCRT, with sequential boost or simultaneous integrated boost approaches being used in our department to treat EC. During the sequential boost approach, the irradiated field encompassed the CTV and the regional lymph node drainage area, with a dose of 41.4–50.4 Gy in 1.8–2-Gy fractions, which was followed by a total RT dose of 50.4–70 Gy to the GTV. During the simultaneous integrated boost approach, VMAT or IMRT was used to simultaneously deliver a high dose (50.4–70 Gy) to the GTV and a lower dose (45–60 Gy) to the CTV and the regional lymph node drainage area. The irradiated tumour length was identified using the CT, barium swallow scans, endoscopy and/or PET-CT. The total RT dose included the doses to the primary tumour from the external beam RT, and patients were grouped according to GTV RT doses of <66 Gy (median: 54 Gy, range: 14.4–65 Gy) or ≥66 Gy (median: 66 Gy, range: 66–70 Gy).

### Chemotherapy

The chemotherapy regimens were selected based on the patient’s age, general physical condition, and the oncologist’s preference. Most patients (n = 95) received cisplatin 60–100 mg/m^2^ on day 1 plus 5-fluorouracil (5-FU) 500–1000 mg/m^2^ daily on days 1–4 through continuous intravenous infusion every 3–4 weeks. Two cycles of cisplatin plus 5-FU were administered concurrently during RT. The other regimens during RT were cisplatin monotherapy (n = 14) with 30–40 mg/m^2^ weekly or 60–100 mg/m^2^ every 3–4 weeks, and 5-FU monotherapy (n = 6) with 500–1000 mg/m^2^ daily on days 1–4 every 3–4 weeks. Patients received additional maintenance chemotherapy if a medical oncologist determined that their medical condition and disease status would allow this.

### Follow-up and toxicity assessment

Toxicity assessments were performed weekly during the RT. The first follow-up evaluation was performed at 1 month after RT and follow-ups were then continued at 3–6-month intervals. During the follow-up, the patients underwent physical examinations, blood and biochemical testing, chest CT, barium scans, and abdominal sonography. Upper gastrointestinal panendoscopy with biopsy or PET-CT were performed if clinically indicated. Toxicities were scored using version 4.0 of the Common Terminology Criteria for Adverse Events, and toxicities were considered acute if they developed within 1 month after completing RT.

### Statistical analysis

The OS interval was calculated from the start of RT to death or the last follow-up. The DPFS interval was calculated from the start of RT to the first instance of recurrence, death, or the last follow-up. The LPFS and DMPFS intervals were calculated from the start of RT to the last follow-up or the first instance of local or distant recurrence, respectively. Categorical variables were analysed using the chi-square test, and survival analyses were performed using the Kaplan-Meier method. Univariate and multivariate analyses were performed using the log-rank test and a Cox proportional hazard regression model, respectively. A Cox proportional hazards model was used to determine the HR and 95% CI values. Differences were considered statistically significant at a p-value of <0.05. All analyses were performed using SPSS software (version 18.0; SPSS, Chicago, IL).

## Data Availability

The datasets generated during and/or analysed during the current study are available from the corresponding author on reasonable request.
